# Dose calculation with brief-pulse ECT demystified

**DOI:** 10.4103/0019-5545.70995

**Published:** 2010

**Authors:** Chittaranjan Andrade

**Affiliations:** Department of Psychopharmacology, National Institute of Mental Health and Neurosciences, Bangalore - 560 029, India

There are two important categories of electroconvulsive therapy (ECT) devices: constant voltage, sinusoidal wave devices and constant current, brief-pulse devices. The sinusoidal wave devices are currently considered obsolete for the following reasons.[1]

1. Current flows almost continuously with the sinusoidal waveform. As a result, far more electrical charge is delivered than is necessary to trigger the seizure. The extra charge may not increase efficacy but does increase the cognitive adverse effects of the treatment. In contrast, with constant current, brief-pulse ECT devices, current is delivered in short pulses. The seizure which is triggered can be as effective as that with sinusoidal wave ECT, and is associated with less cognitive adverse effects.
2. According to Ohm’s law, the current in a circuit is inversely proportional to the resistance. As resistance varies from patient to patient, at constant voltage the current in the circuit and hence the electrical charge delivered during ECT will also vary from patient to patient. Consequently, there is no way that a clinician can deliver a planned ECT dose (in units of charge) to the patient; this, however, is possible with constant current devices which automatically adjust the strength of the current to the resistance in the circuit.

The administration of a planned dose is necessary because it is now known that the electrical dose administered during ECT is just as important to therapeutics as the dose of a drug administered during pharmacotherapy. What is the relationship between dosing and outcome with ECT? There are three fairly consistent findings:[1]

1. Higher electrical doses are associated with a greater proportion of responders in patients who receive right unilateral ECT.
2. Higher electrical doses are associated with faster response to ECT with both unilateral and bilateral electrode placements.
3. Higher electrical doses are associated with greater cognitive adverse effects.

Dosing is usually estimated in units of charge (millicoulombs), although both units of energy (joules) and power (watts) have been suggested as alternatives. The ideal unit remains elusive;[2] nevertheless, for most practical purposes, units of charge suffice.

Many clinicians feel intimidated by the choices available with brief-pulse apparatuses and lack the knowledge necessary to calculate the ECT dose. This article demystifies the subject; all the background that the reader requires is a very basic knowledge of mathematics and physics.

Consider the following facts:

1. Brief-pulse ECT delivers a train of identical pulses of electricity [Figure 1].
2. Each pulse has certain amplitude (pulse height). This is measured in units of current; that is, amperes (A) or milliamperes (mA).
3. Each pulse has certain duration (pulse width). This is measured in milliseconds (ms).
4. There is a specific number of pulses delivered each second. This is determined from the stimulus frequency, which is measured in hertz (Hz) or cycles per second (cps). If the stimulus is unidirectional, the number of pulses per second is the same as the stimulus frequency. If the stimulus is bidirectional, the number of pulses per second is double the stimulus frequency (this is because each cycle is made up of one positive and one negative pulse). Most constant current, brief-pulse ECT devices deliver bidirectional pulses.
5. The ECT stimulus is passed for a specific duration. This is known as the stimulus duration, or the duration of the stimulus train, and is measured in seconds.

**Figure 1:**
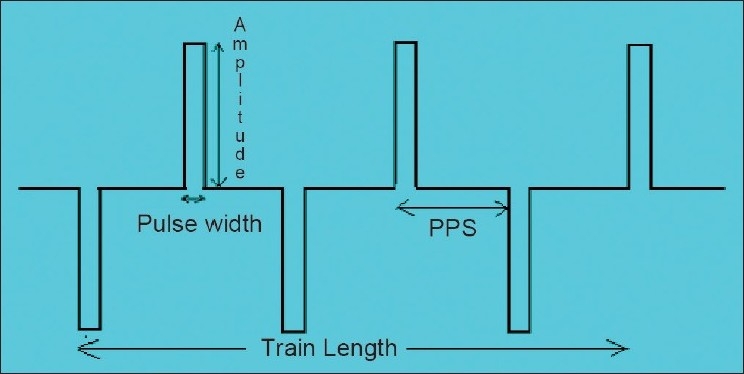
Brief-pulse ECT waveform and components thereof

## Calculating stimulus dose

### Step 1:

To calculate the ECT dose, the first step is to determine the following:

1. Current, or pulse amplitude (A or mA)
2. Pulse width (ms)
3. Number of pulses per second (determined from the stimulus frequency)
4. Stimulus duration

Some ECT devices allow the clinician to adjust the value of all these settings. Other devices have certain of these settings fixed, and certain of these settings under the clinician’s control. The manual of the device and its control panel usually provide the necessary information about the values of the settings that are fixed, and the values of the settings that the clinician can manipulate.

In the event that the clinician is unable to determine any of the above values from the manual, the control panel, and the manufacturer, a visit to any electronics establishment which owns an oscilloscope can quickly and accurately determine not only the values of the fixed and variable parameters but also the nature of the stimulus delivered by the instrument and the fidelity of the settings. Clinicians would be wise to check the fidelity of their instruments periodically, such as every 6-12 months, even if they know all the values.[3]

### Step 2:

The second step is to calculate the number of pulses delivered. This is done as follows:

Number of pulses delivered = (number of pulses per second) × (stimulus duration in s).

### Step 3:

The third step is to calculate the total time for which current is flowing. This is done as follows:

Total time in ms = (pulse width in ms) × (number of pulses delivered)

### Step 4:

The fourth and final step is to calculate the ECT dose that is set, measured in units of electrical charge; that is, millicoulombs (mC). The dose is calculated using the formula:

Charge = current × time.

Thus,

Dose in mC = (current in A) × (total time in ms).

Worked example

Calculating the ECT dose is actually far easier than it may appear from the preceding instructions. Here is a sample calculation for an ECT device which delivers bidirectional pulses with the following settings:

Pulse amplitude (current) = 800 mA (that is, 0.8 A)

Pulse width = 1.2 ms

Stimulus frequency = 60 Hz

Stimulus duration = 2 s

Since the apparatus delivers 60 bidirectional pulses per second, the total number of pulses per second is 60 × 2 = 120.

The number of pulses delivered during the 2 second stimulus is 120 × 2 = 240.

The total time for which current flowed is 240 × 1.2 = 288 ms.

The charge delivered is 0.8 × 288 = 230.4 mC.

## Some practical recommendations for brief-pulse ECT are:

1. Set pulse width at 0.5-1.0 ms
2. Set pulse frequency at 100-200 pulses per second
3. Set pulse amplitude at 0.5-1.0 A
4. Set stimulus duration at whatever length is necessary to create the charge that is desired. Stimulus duration can be set anywhere between 0.2 and 4 s; stimuli that are 4-8 s in duration may also be occasionally administered.

## What dose should a clinician choose?

Most patients have an initial seizure threshold of about 50-100 mC. The threshold may be lower or higher, depending on a number of variables.[1] Clinicians should choose stimulus settings in this range for the initial stimulus if they wish to administer threshold ECT, and stimulus settings at higher values if they wish to administer suprathreshold ECT. Usually, doses that are 50-100% suprathreshold are sufficient for bilateral ECT; thus, if the threshold is 75 mC, a dose of 110-150 mC should yield clinically satisfactory results. Recent research suggests that doses with unilateral ECT should be at least 400 mC in magnitude, or 5-8 times suprathreshold. Other guidelines have also been provided. These are beyond the scope of the present article.

It may be noted that, when increasing the ECT dose, an increase in stimulus duration is usually the best way to increase the charge of the stimulus.[4,5]

Here are two exercises for practice

1. An instrument delivers bidirectional pulses. Current is set at 800 mA. Pulse width is set at 0.75 ms. Stimulus frequency is set at 70 Hz. Stimulus duration is set at 0.75 s. What is the electrical charge delivered?
2. An instrument delivers unidirectional pulses. Current is set at 500 mA. Pulse width is set at 1 ms. Stimulus frequency is set at 80 Hz. Stimulus duration is set at 4 s. What is the electrical dose delivered?

(Answer, 63 mC).

(Answer, 160 mC).
